# Retention in intensive adherence counselling as a pathway to viral suppression among virally non-suppressed adolescents and young people living with HIV in east-central Uganda: a sequential explanatory mixed-methods study

**DOI:** 10.21203/rs.3.rs-10075410/v1

**Published:** 2026-07-25

**Authors:** David Livingstone Ejalu, Peter Simon Okello, Joan Nangendo, Achilles Katamba, Anne R. Katahoire, Sabrina Bakeera-Kitaka, Joan Kalyango, Adithya Cattamanchi, Fred C. Semitata, Moses R. Kamya

**Affiliations:** Makerere University; Makerere University; Makerere University; Makerere University; Makerere University; Makerere University; Makerere University; University of California, Irvine; Makerere University; Makerere University

**Keywords:** intensive adherence counselling, retention, viral load suppression

## Abstract

**Background:**

Intensive Adherence Counselling (IAC) is the recommended intervention for people living with HIV who experience virological non-suppression. However, evidence on retention across the IAC cascade and factors influencing sustained engagement among adolescents and young people living with HIV (AYPLHIV) remains limited. We assessed retention in IAC, its associated factors, and its relationship with viral load suppression among virally non-suppressed AYPLHIV in east-central Uganda.

**Methods:**

We conducted a sequential explanatory mixed-methods study across 32 public health facilities. Quantitative data were retrospectively extracted for 580 virally non-suppressed AYPLHIV aged 10–24 years enrolled in IAC between 2019 and 2024. Retention was defined as completion of all recommended IAC sessions. Modified Poisson regression with robust standard errors identified factors associated with retention. Twelve purposively selected participants, including AYPLHIV, caregivers, and healthcare providers, participated in in-depth interviews. Qualitative data were analysed using a deductive thematic approach guided by the Capability, Opportunity, Motivation-Behaviour framework.

**Results:**

Participants were predominantly female (62.8%) with a median age of 16.4 years (IQR: 12.9–21.2). Retention declined progressively across the IAC cascade from 100% at initiation to 86.7% at IAC-1, 71.2% at IAC-2, and 64.8% at IAC-3. Participants who completed all IAC sessions achieved significantly higher viral suppression than those who did not complete the cascade (p < 0.001). Retention was higher among participants receiving peer support (aPR = 1.35, 95% CI: 1.17–1.55), counsellor-led IAC (aPR = 2.80, 95% CI: 1.53–5.13), and those living within 5 km of a facility (aPR = 1.22, 95% CI: 1.01–1.49). Lower retention was associated with difficulty coping with HIV status, disruption of differentiated service delivery pathways, treatment supporter involvement and VHT support. Qualitative findings attributed disengagement to emotional distress, transport barriers, and substitution of formal counselling by family and community support systems.

**Conclusions:**

Retention in IAC among virally non-suppressed AYPLHIV was suboptimal and strongly associated with viral load suppression. Strengthening peer support, psychosocial care, counsellor-led services, and integration of IAC within differentiated HIV service delivery models may improve retention and treatment outcomes among AYPLHIV.

## Introduction

Adolescents and young people living with HIV (AYPLHIV) remain a disproportionately affected and underserved population within the global HIV response. Recent estimates indicate that approximately 6.75 million adolescents and young people are living with HIV globally, with nearly 80% residing in sub-Saharan Africa [1, 2]. In Uganda, where approximately 1.4 million people are living with HIV. AYPLHIV contribute substantially to new HIV infections and experience poorer treatment outcomes with nearly 38,000 new infections reported annually and about 40% occurring within this age group [2–4]. Despite expanded access to antiretroviral therapy (ART), viral load suppression among AYPLHIV remains considerably lower than among adults. Nationally representative data indicate persistently suboptimal viral suppression among younger populations, with some regions reporting suppression rates below national targets, while districts in east-central Uganda continue to report viral load suppression as low as 39% among the young population [4, 5].

Within the HIV care cascade, successful viral suppression depends not only on access to ART but also on sustained engagement with adherence support interventions designed to address treatment failure [6–8]. Among individuals with virological non-suppression, the Uganda Ministry of Health (MOH) recommends Intensive Adherence Counselling (IAC), a structured intervention comprising at least three client-centred counselling sessions delivered one month apart over a minimum of three months (MOH, 2020). The IAC intervention aims to identify and address barriers to adherence before treatment modification is considered. However, the effectiveness of IAC is inherently dependent on retention throughout the counselling cascade. Retention in IAC represents a critical intermediate implementation outcome because participants must complete the intervention before its intended effects on adherence and viral suppression can be realized [9–11]. Evidence from Uganda and other African settings demonstrates substantial attrition across the IAC cascade, with many clients failing to complete all recommended counselling sessions [9, 11].

Despite the central role of retention in determining IAC effectiveness, factors influencing continued engagement remain poorly understood. Existing studies have largely focused on adherence levels, viral suppression, and clinical outcomes, with comparatively limited attention to retention as a distinct implementation outcome and the mechanisms that facilitate or hinder participation throughout the counselling process [11–13]. Therefore, this study fills this gap by determining the level of retention across the IAC cascade and its relationship to viral load suppression. The secondary objective was to identify individual, treatment-related, and health system factors associated with retention. Using a sequential explanatory mixed-methods approach, we sought to generate evidence to inform adolescent-responsive IAC delivery strategies that strengthen engagement, improve retention, and ultimately enhance viral suppression outcomes.

## Methods

### Study Design

We conducted a sequential explanatory mixed-methods study with a quantitative priority (QUAN→qual) approach. The initial quantitative phase involved retrospective analysis of routine program data to determine retention across the IAC cascade and identify associated factors. Quantitative findings subsequently informed a qualitative descriptive phase that was guided by the Capability, Opportunity, Motivation-Behaviour (COM-B) framework to explore mechanisms underlying observed associations. Integration occurred at interpretation stages. Quantitative findings informed purposive selection of qualitative participants, while qualitative themes were used to explain statistically significant quantitative associations. Integrated findings were presented using a joint display matrix linking quantitative results to COM-B domains and explanatory themes, consistent with Good Reporting of Mixed Methods Studies (GRAMMS) recommendations [14].

### Study Setting

The study was conducted in 32 public ART-providing health facilities located in Jinja City, Jinja, Mayuge, and Kamuli districts in east-central Uganda. The facilities included Health Centre III (sub-county level), Health Centre IV (county level), and general hospital (district level) serving predominantly rural and peri-urban populations, with established HIV clinics delivering IAC according to Uganda Ministry of Health guidelines [15]. These districts were purposively selected because surveillance and programmatic data from the Uganda Population-based HIV Impact Assessment (UPHIA) and district HIV program reports consistently demonstrated suboptimal viral suppression and challenges in achieving the third UNAIDS 95-95-95 target among adolescents and young people. Viral load suppression in this region is as low as 39.3% among children aged 0–14-years and 39.6% among Young People aged 20–24 years [4, 5].

### Study Population

The quantitative component included AYPLHIV aged 10–24 years with documented virological non-suppression who were enrolled in IAC between January 2019 and December 2024. Eligible participants were required to have documented records on IAC initiation, counselling attendance, appointment adherence, and follow-up viral load monitoring available in electronic medical records or paper-based clinical records. Participants who transferred out, died before completing IAC, or had incomplete follow-up information were excluded. For the qualitative component, AYPLHIV were purposively selected from the quantitative cohort to represent diverse retention experiences, including retained and non-retained participants. Caregivers and healthcare providers directly involved in IAC delivery, including counsellors, nurses, and clinical mentors, were also recruited to provide complementary perspectives on factors influencing retention.

### Sample Size Determination

For the quantitative component, the sample size was calculated to detect meaningful differences in retention among AYPLHIV enrolled in IAC using methods for binary outcomes [16, 17]. Estimates were informed by previous studies showing variability in adolescent adherence and retention in IAC, with approximately 80% achieving optimal adherence and engagement in care [9, 11, 12]. To account for clustering within health facilities, a design effect of 2 was applied in line with recommendations for cluster-based studies [18, 19]. Assuming 95% confidence and 80% power, the minimum sample size was estimated at 580 participants. For the qualitative phase, sample adequacy was guided by theoretical sufficiency within the COM-B framework. Data collection continued until no new concepts emerged within the Capability, Opportunity, or Motivation domains and additional interviews no longer enhanced interpretation of quantitative findings. This threshold was achieved after 12 interviews and documented through ongoing analytic memoing and team debriefing.

### Sampling procedure.

Facility-specific sampling frames were constructed by identifying all eligible AYPLHIV enrolled in IAC during the study period. The total sample was proportionately allocated to each facility according to the number of eligible participants. Within facilities, participants were selected using simple random sampling, whereby unique identification numbers were assigned to those eligible and random numbers generated in STATA were used to select study participants. For the qualitative phase, maximum variation purposive sampling was used to recruit AYPLHIV with differing retention outcomes, ages, sexes, residence, schooling status, and distances from health facilities. Caregivers and healthcare providers were subsequently recruited through facility HIV clinics based on their involvement in IAC implementation and follow-up activities.

### Data Collection

Quantitative data were extracted from electronic medical records, Ministry of Health viral load registers (HMIS ACP 001), and HIV care cards (HMIS ACP 003) using a structured Open Data Kit (ODK) tool designed to capture demographic, psychosocial, clinical, treatment-related, and health system characteristics. Research assistants experienced in HIV program monitoring underwent standardized training, and the data collection tool was piloted among 20 non-participants before implementation. Data quality was maintained through built-in validation checks, mandatory fields, routine supervision, and regular verification by the principal investigator. The qualitative phase involved in-depth semi-structured interviews guided by a COM-B-informed interview guide exploring experiences with IAC and factors influencing retention (provided as supplementary material). Interviews were conducted in Lusoga, Luganda, or English by trained trilingual research assistants in private settings, audio-recorded with consent, transcribed verbatim, translated where necessary, and verified against source recordings for accuracy.

### Variable Definitions

The primary outcome was retention in IAC, defined as completion of all scheduled counselling sessions within 4 months of IAC initiation, before repeat viral load testing. This definition was based on the Uganda Ministry of Health guidelines, which recommend three monthly counselling sessions completed within three months, while allowing an additional month to accommodate routine programmatic variations in appointment scheduling [15]. Peer support was defined as the receipt of adherence or psychosocial support from trained adolescent peer supporters or Young Adolescent Program (YAP) members during IAC. Treatment supporter involvement referred to documented support from a caregiver, family member, or designated treatment supporter. Village Health Team (VHT) support referred to adherence follow-up or counselling provided by community health workers. Difficulty coping with HIV status was defined as documented self-reported emotional distress, non-acceptance of HIV status, or psychosocial challenges affecting engagement in care. All other explanatory variables were selected using a conceptual framework informed by the WHO framework of adherence [20].

### Data Analysis

Quantitative data were analysed using STATA version 17.0 (StataCorp, College Station, TX, USA). Descriptive statistics were used to summarize participant characteristics and retention outcomes. Relationship between retention in IAC and viral suppression was determined using the chi-square test. Associations between explanatory variables and retention were assessed using modified Poisson regression with robust standard errors while accounting for clustering at facility level. Variables with p < 0.05 in bivariate analysis were included in a multivariable model built using backward elimination. Confounding was assessed by comparing crude and adjusted estimates, with a ≥ 10% change indicating confounding. Multicollinearity was evaluated using variance inflation factors, interaction terms were included in the model to assess effect modification using likelihood ratio tests, while model fitness was evaluated using the Hosmer-Lemeshow goodness-of-fit test. Age and sex were retained in all models irrespective of significance because of their established relevance to adolescent HIV outcomes. Adjusted prevalence ratios (aPRs) and 95% confidence intervals are reported, with statistical significance set at p < 0.05.

Qualitative data were analyzed using data using a deductive thematic approach guided by a predefined COM-B coding framework. Coding was conducted independently by the principal investigator and an experienced qualitative researcher, with discrepancies resolved through consensus. Themes were organized within the Capability, Opportunity, and Motivation domains and used to explain significant quantitative findings. Quantitative and qualitative findings were integrated using a following-a-thread approach, whereby statistically significant quantitative findings were identified as initial threads and subsequently explored within the qualitative data to understand the behavioural, psychosocial, and health system mechanisms underlying observed associations. Themes derived from the COM-B framework were then used to explain, expand, and contextualize quantitative results. To enhance reflexivity, interviews were conducted by trained research assistants independent of participants’ clinical care, while the research team engaged in ongoing reflection regarding potential influences on data collection, analysis, and interpretation.

## RESULTS

### Participant demographic characteristics

A total of 580 virally non-suppressed AYPLHIV enrolled in IAC between 2019 and 2024 were included. Participants were predominantly female (62.8%, n = 364), with a median age of 16.4 years (IQR: 12.9–21.2). Nearly half (43.5%, n = 252) were aged 10–14 years. Most participants had received ART for 1–4 years (62.9%, n = 365), were receiving first-line ART (89.3%, n = 518), resided in rural areas (74.8%, n = 434), and were enrolled in school (64.7%, n = 375) (Table 1).

#### Retention of non-suppressed AYPLHIV in IAC in east-central Uganda.

**Figure 1:**
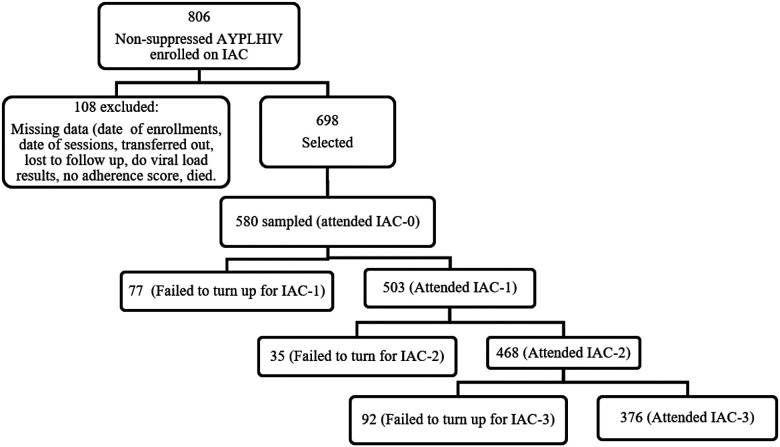
Retention cascade across IAC sessions

Retention declined progressively across the IAC cascade (Fig. 1). All participants initiated IAC (580/580, 100%), but attendance declined to 503/580 (86.7%) at IAC-1, 413/580 (71.2%) at IAC-2, and 376/580 (64.8%) at IAC-3. Overall, 35.2% of AYPLHIV failed to complete the full counselling cascade. The largest absolute loss occurred between IAC-1 and IAC-2 (n = 90), highlighting the second counselling session as a critical point of disengagement.

### Viral load suppression across the IAC cascade

Viral load suppression generally increased with retention across the IAC cascade as shown in Table 2. Among participants who attended one IAC session, those retained were significantly more likely to achieve viral suppression than those not retained (56.7% vs. 36.4%, p = 0.001). Overall, participants who completed all recommended IAC sessions had a significantly higher viral suppression rate compared with those who did not complete the IAC cascade (59.6% vs. 43.6%, p < 0.001).

### Factors associated with retention in IAC

In multivariable analysis (Table 3), retention was significantly higher among participants receiving counsellor-led IAC (aPR = 2.80, 95% CI: 1.53–5.13), peer support during IAC (aPR = 1.35, 95% CI: 1.17–1.55), and those residing within 5 km of a health facility (aPR = 1.22, 95% CI: 1.01–1.49). Lower retention was observed among participants reporting difficulty coping with their HIV status (aPR = 0.56, 95% CI: 0.40–0.79), receiving support from Village Health Teams (aPR = 0.64, 95% CI: 0.42–0.97), having a home treatment supporter (aPR = 0.80, 95% CI: 0.69–0.94), receiving bimonthly ART refills before IAC initiation (aPR = 0.45, 95% CI: 0.20–0.97), and those previously enrolled in Community Drug Distribution Points (aPR = 0.77, 95% CI: 0.63–0.93).

### Qualitative findings

Six interrelated themes were identified across the Capability, Opportunity, and Motivation constructs that interact to influence sustained engagement in IAC.

Table 4 summarizes the demographic profile and facility distribution of participants. Twelve interviews were conducted with participants drawn from Health Centre III, Health Centre IV, and hospital settings across both rural and urban locations. AYPLHIV included virally suppressed, non-suppressed, retained and non-retained individuals. All caregivers and health workers were aged 31–53 years.

### Capability

#### Theme: Emotional distress and challenges with HIV status acceptance

Emotional distress and unresolved acceptance of HIV status emerged as major barriers to psychological capability and provided a clear explanation for the lower retention observed among participants reporting difficulty coping with their HIV status. Participants described feelings of hopelessness, emotional exhaustion, and uncertainty about the future, which reduced their ability to engage consistently with counselling appointments. One adolescent explained:

“Sometimes I feel like I have no future…this kind of life is hard. Even attending those sessions is not easy.”

(Young adult, 22 years, non-retained non-suppressed, rural facility).

A counsellor similarly noted:

“We have noticed that those who are distressed and depressed find it so hard to comply with the instructions we give during IAC. We have to put in much effort to follow up and send reminders; otherwise, they never turn up.”

(Counsellor, 11 years’ experience, rural facility).

### Opportunity

#### Theme: Provider-client interaction

Provider-client interaction emerged as an important social opportunity factor positively or negatively influencing retention depending on how it is perceived. Participants consistently described counsellors as more approachable, empathetic, and attentive than nurses. AYPLHIV reported that positive interactions encouraged attendance, whereas unsupportive encounters discouraged continued participation. These findings provide a plausible explanation for the significantly higher retention observed among participants receiving counsellor-led IAC. One participant stated:

“When a counsellor talks to you, they listen, they ask about your life, and they help you plan. Nurses are in a rush, and sometimes they scold you if your results are bad.”

(Adolescent, 13 years, retained non-suppressed, rural facility).

Another participant reported:

“I didn’t attend the last two sessions because I didn’t find the counsellor around; the nurse who was on duty does not do me well…”

(Adolescent, 17 years, retained suppressed, urban facility).

#### Theme: Distance and transport constraints

Physical accessibility emerged as an important determinant of retention. Participants living near health facilities described fewer financial and logistical barriers to attendance, whereas those residing farther away frequently struggled with transport costs and travel time. These accounts explain the higher retention observed among adolescents residing within five kilometres of a health facility. One participant explained:

“It’s easy for me to walk to the clinic. It is near home. I go early and return early. I don’t miss any sessions anyhow.”

(Adolescent, 17 years, retained suppressed, urban facility).

Providers highlighted the challenges faced by those living farther away:

“Transport is a big problem to those far away, they can’t walk to the facility for counselling and most of them lack support, especially those with no parents.”

(Clinical mentor, 13 years’ experience, urban facility).

#### Theme: Family and community-level support as a substitution mechanism

Although treatment supporters and VHTs were intended to strengthen adherence support, participants described situations in which these support systems inadvertently substituted for formal facility-based counselling. This theme provides a potential explanation for the unexpected finding that treatment supporter and VHT involvement were associated with lower retention. One caregiver stated:

“I kept on encouraging and supporting him to take the medicine so that he would get suppressed. Even if he attended those sessions, they would still tell him the same thing…take the drug… take the drug on time”

(Caregiver, 39 years old, 14 years on ART, urban facility).

A counsellor similarly observed:

“We have recorded cases where parents and family members want to act like counsellors… the adolescent will tell you that my mummy or my aunt or the VHT talked to me about taking the meds regularly, so I saw no need to waste money on transport coming for counselling”

(Counsellor, 15 years’ experience, urban facility).

#### Theme: IAC delivery requirements and disruption of routine service delivery

The structure of IAC delivery emerged as a physical opportunity barrier. Participants who were accustomed to longer refill intervals or community-based HIV service delivery perceived the transition to monthly facility attendance during IAC as burdensome. Increased transport costs, work commitments, school schedules, and concerns about disclosure made frequent weekday attendance difficult. These experiences help explain the lower retention observed among participants previously receiving bimonthly refills or those in differentiated service delivery (DSD) models. One young person stated:

“I used to get drugs to last for many months, and it was okay, but this kind of counselling needed me to attend the clinic every month, which was not easy for me… I couldn’t frequently ask for permission off work; they would become suspicious”

(Adolescent, 19 years, non-retained, suppressed, rural facility).

Health workers also described resistance among adolescents transitioning from decentralized care models:

“We have some cases where these non-suppressed young people, whom we give care at the community level, do not want to come to the facility for IAC for various reasons, including fear and stigma…whenever we have fuel, we follow them up to the community”

(Nurse, 5 years’ experience, rural facility).

### Motivation

#### Theme: Peer support

Peer support emerged as an important source of reflective motivation that encouraged continued participation in IAC. Participants described peers as role models whose successful treatment experiences inspired hope, strengthened self-belief, and reinforced commitment to counselling attendance. Health workers similarly noted that peer-led support helped sustain motivation and engagement among vulnerable adolescents. These findings explain the higher retention observed among participants who received peer support during IAC. One adolescent explained:

“The YAP gave me hope. I saw someone like me doing well, and I wanted to be like them. That’s when I started keeping appointments for the counselling sessions seriously”.

(Adolescent, 16 years, retained suppressed, urban facility).

Health workers corroborated this observation:

“We have used peers for some time now, and they have really helped us to keep track and motivate these fragile groups of young patients to not only keep their appointments but also to adhere.”

(Nurse, 11 years’ experience, urban facility).

Table 5 integrates quantitative and qualitative findings using the COM-B framework. Retention in IAC was influenced by psychological capability, social and physical opportunity, and reflective motivation. Emotional distress reduced engagement, while counsellor-led care, peer support, and proximity to health facilities promoted retention through trust, motivation, and reduced access barriers. Conversely, treatment supporter and VHT involvement sometimes substituted for formal counselling, and increased visit requirements disrupted engagement.

## Discussion

This sequential explanatory mixed-methods study assessed retention among virally non-suppressed AYPLHIV enrolled in IAC in east-central Uganda and explored factors influencing sustained engagement. Integration of these findings followed a thread-based approach in which statistically significant quantitative associations were traced through qualitative narratives. This process demonstrated that observed retention patterns were explained by interacting capability, opportunity, and motivation mechanisms operating at individual, social, and health system levels. Retention across the cascade was suboptimal, with only 64.8% of participants completing the recommended counselling sessions. Importantly, retention was strongly associated with viral load outcomes, as participants who completed the full IAC cascade achieved significantly higher viral suppression than those who did not (59.6% versus 43.6%, p < 0.001). These findings suggest that retention may represent a critical implementation outcome through which IAC exerts its effect on viral suppression. The retention estimates observed in this study are consistent with reports from Uganda, Kenya, Tanzania, Nigeria and other African settings documenting substantial attrition across enhanced adherence counselling cascades [9, 10, 13, 21]. Similarly, the higher viral suppression observed among participants retained throughout IAC aligns with evidence showing that completion of enhanced adherence counselling is associated with improved virological outcomes among adolescents and adults living with HIV [22, 23]. From an implementation science perspective, these findings reinforce the importance of retention as an intermediate implementation outcome that links intervention delivery to clinical effectiveness.

One notable finding was the association between peer support and improved retention. Participants receiving peer support were significantly more likely to complete IAC, and qualitative findings showed that peers enhanced motivation through role modelling, treatment optimism, and shared lived experiences. Similar findings have been reported among AYPLHIV across sub-Saharan Africa, where peer-led interventions improve engagement, adherence, and treatment outcomes [21, 24, 25]. Likewise, adolescents counselled by dedicated counsellors demonstrated higher retention than those counselled by nurses. Qualitative findings suggested that counsellors were perceived as more approachable and responsive to adolescents’ psychosocial needs. These findings indicate that the quality of interpersonal interactions may be as important as the counselling content itself, highlighting the importance of adolescent-responsive service delivery models.

An unexpected finding from our study was that treatment supporter involvement and VHT support were associated with lower retention. Conventional HIV care models assume that social support invariably promotes adherence and engagement [24, 26]. Our qualitative findings suggested that family members and community health workers occasionally substituted for formal counselling, reducing the perceived need for facility attendance. However, alternative explanations should also be considered. Adolescents receiving VHT or treatment supporter involvement may have represented a higher-risk subgroup with greater adherence challenges, psychosocial vulnerabilities, or prior disengagement from care. Residual confounding, referral bias, and unmeasured differences in severity may partially explain these associations. Consequently, the findings should not be interpreted as evidence that family or community support reduces retention, but rather that current integration between community-based support systems and formal IAC delivery may be suboptimal.

Participants reporting difficulty coping with their HIV status were substantially less likely to remain engaged in IAC. Qualitative findings revealed that emotional distress, self-stigma, and poor acceptance of HIV status reduced psychological readiness to participate in counselling. These findings are consistent with studies from Uganda, Tanzania, Ghana, Botswana, and Kenya demonstrating that psychosocial distress undermines adherence, retention, and viral suppression among adolescents living with HIV [21, 27–29]. The findings suggest that psychosocial support should be considered an integral component of IAC rather than a parallel intervention.

Geographical accessibility and service delivery design also influenced retention. Adolescents living closer to facilities were more likely to complete IAC, while those transitioning from community-based DSD models to monthly facility-based counselling experienced lower retention. Similar observations have been reported in Uganda and other African settings where DSD models improve retention by reducing travel burden and opportunity costs [12, 30–32]. The findings suggest that requiring adolescents to revert from community-based ART delivery to intensive facility-based counselling may unintentionally recreate barriers that DSD models were designed to overcome. Future implementation strategies should explore community-based and hybrid IAC models capable of maintaining counselling intensity while preserving the accessibility benefits of DSD.

### Strengths and Limitations

This study combined quantitative programmatic data with qualitative inquiry within a sequential explanatory mixed-methods design, enabling a comprehensive assessment of retention and the mechanisms influencing engagement in IAC. Application of the COM-B framework strengthened interpretation of behavioural, psychosocial, and structural determinants, while inclusion of participants from multiple facilities enhanced the programmatic relevance of the findings. However, several limitations should be considered. The observational and retrospective design limits causal inference and raises the possibility of residual confounding from unmeasured psychosocial, household, and facility-level factors. Selection bias may have occurred because participants with incomplete records, transfers, deaths, and loss to follow-up were excluded. In addition, interviews were conducted after completion of the IAC cascade and may have been subject to recall bias. Finally, integration occurred at the interpretation stage, and alternative explanations for some quantitative associations may not have been fully captured. Therefore, findings should be interpreted within the context of similar public-sector HIV programmes in Uganda and comparable settings.

## Conclusion

Retention in IAC among virally non-suppressed AYPLHIV in east-central Uganda remains suboptimal and is associated with lower viral suppression. Sustained engagement in IAC is influenced by the interplay among psychosocial well-being, provider-client relationships, peer support, geographical access, and service delivery design. These findings suggest that improving viral suppression among non-suppressed AYPLHIV requires strengthening retention as a core implementation outcome of IAC. The Uganda Ministry of Health should consider integrating routine psychosocial screening and structured peer-support mechanisms within IAC services. In addition, adapting IAC delivery to align with differentiated service delivery models through community-based, outreach, or hybrid approaches may reduce disengagement among high-risk adolescents. Future implementation research should evaluate the effectiveness, acceptability, and scalability of these adolescent-centred approaches in routine HIV programmes.

## Supplementary Files

This is a list of supplementary files associated with this preprint. Click to download.

- Appendix4interviewguideAYPLHIVEnglishversion.pdf
- Appendix5interviewguideCaretkersEnglishversion.pdf
- APPENDIX6InterviewguideHealthworkers.pdf

## Figures and Tables

**Table 1:** Participant characteristics of non-suppressed AYPLHIV in east-central Uganda

Variable	Category	Frequency (n = 580)	Percent (%)
Sex	Female	364	62.8
	Male	216	37.2
Age	10–14	252	43.5
	15–19	137	23.6
	20–24	191	32.9
Duration on ART	1–4 years	365	62.9
	5–9 years	131	22.6
	10 + years	84	14.5
Residence	Rural	434	74.8
	Urban	146	25.2
Marital status	Married	140	24.1
	Never married	440	75.9
Schooling status	Out of school	205	35.3
	In school	375	64.7
Orphaned and vulnerable	Yes	101	17.4
	No	479	82.6
ART Regimen during IAC	First line	518	89.3
	Second line	62	10.7
Stability status	Stable	473	81.6
	Unstable	107	18.5
ART delivery model before IAC	FBIM	427	73.6
	CDDP	37	6.4
	FBG	83	14.3
	FTDR	33	5.7

Key: FBIM; Facility-Based Individual Management, CDDP; Community Drug Distribution Points, FBG; Facility-Based Group and FTDR; Fast Track Drug Refill

**Table 2:** Viral suppression outcomes among retained versus non-retained participants

			Suppressed		Chi square
IAC session	Retention	Frequency (%)	No	Yes	p-value
One (IAC-1)	No	77(13.3)	49 (63.6)	28 (36.4)	0.001
	Yes	503 (86.7)	218 (43.3)	285 (56.7)	
Two (IAC-2)	No	35(7.0)	19 (54.3)	16 (45.7)	0.175
	Yes	468 (93.0)	199 (42.5)	269 (57.5)	
Three (IAC-3)	No	92(19.7)	47 (51.1)	45 (48.9)	0.064
	Yes	376(80.3)	152 (40.4)	224 (59.6)	
Attended all IAC Sessions	No	204 (35.2)	115 (56.4)	89 (43.6)	< 0.001
	Yes	376 (64.8)	152 (40.4)	224 (59.6)	

**Table 3:** Bivariate and Multivariate analysis of factors associated with retention in IAC among virally non-suppressed adolescents and young people living with HIV in east-central Uganda.

Variable	Category	Retained in IAC		Unadjusted		Adjusted	
		No (%)	Yes (%)	uPR (95%CI)	p-value	aPR (95%CI)	p-value
Sex	Female	143 (39.3)	221 (60.7)	1			
	Male	61 (28.2)	155 (71.8)	1.18(0.96–1.44)	0.104		
Age	10–14	66 (26.2)	186 (73.8)	1			
	15–19	54 (39.4)	83 (60.6)	0.82(0.71–0.95)	0.008		
	20–24	84 (44.0)	107 (56.0)	0.76(0.54–1.07)	0.114		
Distance to facility	> 5km	117 (46.6)	134 (53.4)	1			
	< 5km	87 (26.4)	242 (73.6)	1.38(1.038–1.83)	0.027	1.22(1.01–1.49)	0.045
IAC provider	Nurse	57 (77.0)	17 (23.0)				
	Counsellor	147 (29.1)	359 (70.9)	3.09(1.56–6.11)	0.001	2.80(1.53–5.13)	0.001
Received peer support during IAC	No	166 (38.4)	266 (61.6)				
	Yes	38 (25.7)	110 (74.3)	1.21(1.03–1.42)	0.023	1.35(1.17–1.55)	< .001
Reported challenge coping with status	No	163 (31.5)	355 (68.5)	1			
	Yes	41 (66.1)	21 (33.9)	0.49(0.32–0.76)	0.001	0.56(0.40–0.79)	0.001
Has a home treatment supporter	No	13 (23.2)	43 (76.8)	1		1	
	Yes	191 (36.5)	333 (63.6)	0.83(0.69–0.98)	0.032	0.80(0.69–0.94)	0.005
Received support from VHT	No	158 (31.2)	349 (68.8)				
	Yes	46 (63.0)	27 (37.0)	0.54(0.30–0.95)	0.034	0.64(0.42–0.97)	0.04
Refill frequency before IAC initiation	Monthly	178 (33.0)	362 (67.0)	1			
	Bimonthly	17 (73.9)	6 (26.1)	0.39(0.17–0.90)	0.028	0.45(0.20–0.97)	0.04
	Quarterly	9 (52.9)	8 (47.1)	0.70(0.36–1.36)	0.295	0.84(0.58–1.22)	0.35
ART delivery model before IAC	FBIM	127(29.7)	300(70.3)	1			
	CDDP	20(54.1)	17(45.9)	0.65(0.52–0.82)	< 0.001	0.77(0.63–0.93)	0.007
	FBG	38(45.8)	45(54.2)	0.78(0.53–1.13)	0.185	1.01(0.77–1.33)	0.957
	FTDR	19(57.6)	14(42.4)	0.60(0.35–1.03)	0.064	0.81(0.58–1.13)	0.212

Key: VHT; Village Health Team, FBIM; Facility-Based Individual Management, CDDP; Community Drug Distribution Points, FBG; Facility-Based Group and FTDR; Fast Track Drug Refill.

**Table 4:** Demographic profile of study participants and sampling distribution across facilities

No	Participant	Facility level	Facility location	Duration in ART	Duration in service
1	Adolescent (retained, non-suppressed)	HC III	Rural	13	-
2	Adolescent (retained, suppressed)	HC IV	Urban	16	-
3	Adolescent (retained, suppressed)	Hospital	Urban	1	-
4	Adolescent (suppressed, non-retained)	HC IV	Rural	2	-
5	Young adult (non-retained, non-suppressed)	Hospital	Rural	3	-
6	Caregiver of retained AYPLHIV	HC IV	Urban	14	-
7	Caregiver of non-retained AYPLHIV	HC III	Rural	-	-
8	Nurse	HC III	Rural	-	4
9	Nurse	HC IV	Urban	-	11
10	Clinical mentor	HC IV	Urban	-	13
11	Counsellor	HC IV	Rural	-	11
12	Counsellor	Hospital	Urban	-	15

Key: HC; Health Centre, M; Male, F; Female

**Table 5:** A following-thread mapping of quantitative findings to qualitative themes

Quantitative Finding	COM-B Domain	Qualitative Theme	Mechanism Explaining Retention
Difficulty coping with HIV status (aPR = 0.56)	Capability (Psychological)	Emotional distress and challenges with HIV status acceptance	Psychological distress and poor self-acceptance reduce readiness to engage in IAC
Counsellor-led IAC (aPR = 2.80)	Opportunity (Social)	Provider-client interaction	Trust, empathy, and supportive communication improve attendance and continuity
Living within 5 km of the facility (aPR = 1.22)	Opportunity (Physical)	Distance and transport constraints	Reduced travel burden facilitates attendance.
Home treatment supporter (aPR = 0.80)	Opportunity (Social)	Family and community support as a substitution mechanism	Informal counselling reduces perceived need for facility attendance
VHT support (aPR = 0.64)	Opportunity (Social)	Family and community support as a substitution mechanism	Community support partially substitutes for formal IAC participation.
Bimonthly refill before IAC (aPR = 0.45)	Opportunity (Physical)	IAC delivery requirements and disruption of routine service delivery	Increased visit frequency creates structural barriers
Peer support during IAC (aPR = 1.35)	Motivation (Reflective)	Peer support as a source of treatment motivation	Role modelling, hope, and treatment confidence strengthen commitment to IAC

## Data Availability

All data supporting the findings of this study are available within the paper and its Supplementary Information.

## Abbreviations

IAC: Intensive Adherence Counselling
AYPLHIV: Adolescents and Young People Living with HIV
FBIM: Facility-Based Individual Management
CDDP: Community Drug Distribution Points
FBG: Facility-Based Group
FTDR: Facility-based, fast-track drug refill
CCLAD: Community client-led ART delivery
VHT: Village Health Teams
